# Fatal Borreliosis in Bat Caused by Relapsing Fever Spirochete, United Kingdom

**DOI:** 10.3201/eid1508.090475

**Published:** 2009-08

**Authors:** Nicholas J. Evans, Kevin Bown, Dorina Timofte, Vic R. Simpson, Richard J. Birtles

**Affiliations:** University of Liverpool, Liverpool, UK (N.J. Evans, K. Bown, D. Tomifte, R.J. Birtles); Wildlife Veterinary Investigation Centre, Truro, UK (V.R. Simpson)

**Keywords:** Borrelia, bat, relapsing fever, borreliosis, spirochete, bacteria, vector-borne infections, United Kingdom, letter

**To the Editor:** Tick-borne relapsing fevers caused by members of the genus *Borrelia* have been encountered throughout Africa, Asia, the Americas and, rarely, in southern Europe (*1*). The *Borrelia* species associated with relapsing fevers form a monophyletic group within the genus, although not all members of this group have yet been implicated as agents of human disease. For example, a novel spirochete that is closely related to the relapsing fever agent *Borrelia turicatae* has recently been detected in *Carios kelleyi*, an argasid bat tick (*2*,*3*). We report the discovery of a spirochete causing fatal borreliosis in a bat in the United Kingdom.

The infected bat was a juvenile female *Pipistrellus* species that was found alive but on the ground near the town of Mevagissy in southwestern England in August 2008; despite rehabilitation efforts, it died a few days later. A postmortem examination showed pale skeletal muscles, anemia, excess blood-tinged pleural fluid, a healthy thymus, but enlarged cranial thoracic lymph nodes. The liver was greatly enlarged and mottled, the spleen was also large and unusually dark, and the adrenal glands were enlarged and pale with areas of hemorrhage. The kidneys were pale with a fine speckling pattern over the cortex. Histopathologic examination of the liver showed multifocal necrosis and vacuolation of hepatocytes and infiltration by macrophages. The lungs were congested and infiltrated by inflammatory cells, and large numbers of granulocytes were found in the blood vessels. The spleen showed marked extramedullary hemopoiesis. Tissue sections stained by the Warthin-Starry technique exhibited numerous long, undulating, argophilic bacilli. These organisms were present in large numbers in the liver lesions (Figure), but were also found in the parenchyma of lung and spleen and in blood vessels.

**Figure:**
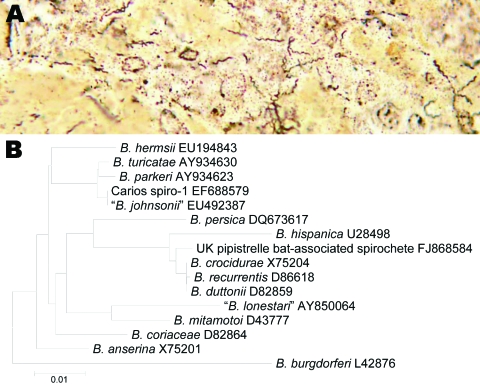
A) Warthin-Starry–stained section of bat liver showing numerous spirochetes. B) Phylogram inferred from 776-bp alignment of *flaB* fragments obtained from infected bat liver tissue and for other members of the relapsing fever group of *Borrelia* species for which sequence data were available. *B. burgdorferi* is included as an outgroup. The numbers appearing after the names of the *Borrelia* species are the relevant GenBank accession numbers. Scale bar indicates nucleotides substitutions per site.

On the basis of these observations, a diagnosis of fatal hepatitis and septicemia caused by a spirochete was made. DNA from the bat’s liver was extracted and analyzed by using a PCR specific for an almost complete fragment of the 16S rRNA-encoding gene, as previously described (*4*), but with an annealing temperature of 45°C. This DNA extract was also incorporated into PCR assays targeting *glpQ* and *flaB* gene fragments (*5*). The products of these reactions were sequenced, and sequence data were assembled and analyzed by using Staden (*6*) and MEGA (*7*).

We obtained unambiguous sequence data for all 3 loci, comprising of 1,364 bp of the 16S rRNA-encoding gene (GenBank accession no. FJ868583), 1,239 bp of *flaB* and flanking regions (GenBank accession no. FJ868584), and 480 bp of *glpQ* (GenBank accession no. FJ868585). Each of these was aligned with homologous sequences available for other *Borrelia* species and used for phylogenetic analyses. Inferences made by using all loci were congruent, with the UK bat–associated spirochete lying close to, but distinct from, a cluster containing *B. recurrentis, B. duttonii,* and *B. crocidurae* (Figure; data not shown).

These 3 species are associated with relapsing fevers in Africa and Asia. The UK bat–associated spirochete bore no specific evolutionary relatedness to *B. johnsonii*, the newly characterized member of the relapsing fever group of *Borrelia* species associated with *C. kellyi* in the United States (Figure) (*3*). An *Argas vespertilionis* larval tick was found attached to the infected bat and may have been the source of its infection. PCR was not performed on the tick because it was near-replete with blood that was intensely infected with spirochetes. *A. vespertilionis*, commonly known as the short-legged bat tick, is widely distributed, parasitizing numerous bat species across Europe, southern Asia, and North Africa (*8*).

Given the close relationship between the novel spirochete we encountered and known pathogens, the reported propensity of *A. vespertilionis* to bite humans (*9*), and the wide geographic range of this tick, our findings have repercussions for public health in many parts of the Old World. Furthermore, although bats are likely the reservoir host for this organism, our study also identifies it as a pathogen, and as such its discovery has implications for the conservation of numerous threatened bat species across Europe and throughout the world.
